# The Determinants of Household Food Waste Generation and its Associated Caloric and Nutrient Losses: The Case of Lebanon

**DOI:** 10.1371/journal.pone.0225789

**Published:** 2019-12-03

**Authors:** Ali Chalak, Mohamad G. Abiad, Mohamad Diab, Lara Nasreddine

**Affiliations:** 1 Department of Agriculture, Faculty of Agricultural and Food Sciences, American University of Beirut, Beirut, Lebanon; 2 Department of Nutrition and Food Sciences, Faculty of Agricultural and Food Sciences, American University of Beirut, Beirut, Lebanon; Universitat Wien, AUSTRIA

## Abstract

Food waste has a great impact on food and nutrition security, the environment, and global, regional as well as national economies. However, little is known about the associated nutrient loss embedded in wasted foods, especially in developing countries, with Lebanon being a case in point. The present paper uses data gathered from a survey of 250 households conducted in Beirut, Lebanon, in which respondents filled 7-day food waste diaries in order to investigate the drivers of food waste generation at the household level and its dietary content. The results show that food waste is approximately 0.2 kg per capita per day in urban Lebanon. This wasted food contains on average 451.2 kcal, 37.5 g carbohydrates, 14.9 g protein, 2.9 g dietary fiber, 2.4 μg vitamin D, 165.2 mg calcium and 343.2 mg potassium. Furthermore, a Tobit analysis of the socio-demographic and behavioral drivers of food waste generation was conducted. This analysis shows that being the sole homeowner, spending more on food, having a larger number of household members, being ready to eat everything prepared, and tending not to buy special offers, significantly increases household food waste generation, at least in terms of physical weight. This is the first study conducted in Lebanon and the Middle East aiming at investigating food waste, and the associated nutrient loss as well as the drivers of food waste generation at the household level. This work could help provide evidence for policymakers to address both food and nutrition security issues in Lebanon.

## 1. Introduction

Food waste and food and nutrition security are gaining an increasing amount of attention among both researchers and policy-makers in both developed and developing countries, all the more so given the growing world population. Food loss and food waste accompany every stage of the food supply chain, with more than 35 percent of overall food losses occurring in both the retail and consumption sectors [1, 2]. While food loss refers to a decrease in mass (dry matter) or nutritional value (quality) of the food that was originally intended for human consumption, food waste refers to food appropriate for human consumption that ends up being discarded, whether it is kept beyond its expiry date or left to spoil [3]. According to the Food and Agriculture Organization (FAO), one-third of all food produced for human consumption is lost or wasted globally [4]. In regions suffering from undernourishment like Africa and South Asia, this immense amount of waste translates into 400 to 500 calories per person per day–and up to 1520 calories in the developed world [5].

Societal costs of wasting food add up to around USD 2.6 trillion globally, of which USD 1 trillion are economic losses to the agro-food chain, USD 900 billion are human welfare losses, and USD 700 billion are losses due to the environmental impact of food waste [6]. In view of all these costs, recent estimates suggest that in 2014–2016 around 795 million people—almost one-ninth of the world population—are undernourished [7]. Minimizing or even eliminating food wastage can help address the dietary needs of one-eighth of the undernourished population globally [8]. There is, therefore, an urgent need to explore various avenues to prevent food waste while contributing to the alleviation of hunger and malnutrition. Indeed there have been many recent examples of private sector initiatives that have successfully gathered food waste to feed undernourished people–an example is Copia, an American startup, more information is available in the following video: https://www.youtube.com/watch?v=to9jcHZyrBY&t=35s.

Interest in food loss and waste (FLW) research has been steadily gaining momentum globally in the last decade. While qualitative FLW research has been focusing mainly on explaining the ways in which societal practices contribute to the passage of food into waste, and conceptualize how multiple factors at different levels interrelate in the contribution to food waste [9], quantitative research has sought to complement this role through assessing the extent to which such interrelations are significant [10, 11].

In the Arab world, although FLW research has increased, in terms of publication output it represents a mere 2.5 percent of the world’s total [12]. Moreover, to our knowledge, very little from this research has been directed towards conducting detailed quantitative assessments of food waste and its implication on diets in the region. Abiad and Meho (2018) attribute this deficiency in research productivity to several possible factors, among which the lack of interest in the subject among local scientists, as well as the lack of funding and governmental support, to conduct it. Lack of interest in this area of research can be at the root of many of the waste crises arising in this part of the world. According to the FAO [13], in the developing world in general and the Middle East and North African (MENA) countries in specific, inadequate data, lack of awareness and technical capacity, inappropriate or even non-existent policies and regulations, institutional and coordination gaps, and underinvestment are some of the most important limitations that have delayed effective action aimed at reducing food losses and waste.

In light of the above, any future attempt to deal with food waste in the MENA region will fail to be evidence-based in the absence of studies that point out the main determinants of food waste. This highlights the importance of conducting research to inform innovative solutions targeting the root micro-level determinants of food waste generation. Research in this vein remains wanting in developing countries, not least as a means to understand how national settings and cultural standards shape food wastage [14] in order to devise effective measures for food waste reduction that are cognizant of regional specificities [15].

One aspect that research on the quantification of food waste has largely overlooked is the assessment of nutrient losses associated with it. Since food waste reduction constitutes an important step towards alleviating undernourishment [16], nutritional evaluation of food waste becomes instrumental in designing food waste reduction schemes with a nutritional mandate [17]. A common approach to nutritional quantification is the expression of food consumption in terms of kilocalories (kcal) per capita per day [18]. This approach was only used in a limited number of studies to estimate the nutritional losses in wasted food [19–21]. However, relying solely on caloric losses could lead to over-representation of the impact of calorie-dense foods whilst ignoring other wasted nutrients. Moreover, the disproportionate waste of nutrient-dense foods like vegetables, fruits, seafood, and dairy products may have an extensive impact on the supply of micronutrients [17]. This has recently generated a new scope for research examining a wider range of nutrients lost due to food waste generation [17, 22].

This paper comes as a first attempt to shed light on the household dimension of food waste generation in Lebanon, a MENA country, using a household field survey that quantifies food waste by means of a weekly food diary. This work aims to quantify, using econometric tools, the socioeconomic, attitudinal and behavioral determinants of household food waste to explore the nutritional implications of food waste in Lebanon.

## 2. Methodology

### 2.1. Overview of potential household food waste determinants

Food waste is a multi-faceted phenomenon, and the study of its drivers calls for the integration of multiple disciplines and cannot be attributed to single causes. Based on the available literature on food waste, these drivers are categorized as follows: (1) concerns, (2) perceptions of food waste, (3) food-related household practices and routines, and (4) sociodemographic characteristics [23]. As the aim of this study is to assess which of the above drive food waste generation in Lebanon, we designed our survey questionnaire with a view to operationalize all of these broad determinants into variables that could be useful in understanding the main roots of food waste generation.

The survey questionnaire tries to cover the concerns of households that may drive them to reduce food waste. One such concern is financial, which has been claimed to rank as the first motivation for reducing food waste heading, while another is environmental [24], though this latter often appears to have a minimal effect on motivating waste reduction behavior probably due to individuals’ lack of awareness and knowledge about the relation between food waste and its ecological impacts [25, 26]. To assess perceptions around food waste, the questionnaire asks a question about guilt feeling, a factor that has been shown to be significant in explaining reduced waste behavior [27].

In terms of food-related practices and routines, strategies like preparing a shopping list are effective tools to reduce food waste as they help to prevent overbuying [27, 28]. Furthermore, appropriate storing practices like ordering and freezing prevent food from going to waste since they enhance the visibility of food in the fridge and extend the shelf-life of food and leftovers [10, 23, 29]. Cooking practices can also play a major role in food waste generation especially when preparing too much food [24], or when having difficulties in predicting portions due to the presence of children at home [30, 31]. Managing leftovers was similarly assessed as it is considered to be an effective tool to decrease food waste [28]. All the above variables have been found to be significant drivers of food waste [23].

In terms of sociodemographic characteristics, research has found variables such as expenditure on food and income to be significant determinants of food waste [2]. When it comes to income, results in the literature are not homogeneous. Some studies found that households with higher income tend to waste more food [32, 33], while others found no correlation between income and food waste [34, 35]. Like income, there is disagreement about the relation between age and food waste generation in the literature. As for age, most studies show that it is negatively correlated with food waste [23, 28, 35], which seems to indicate greater frugality and knowledge of food waste consequences among older people [11]. Finally, household size was found to be an important variable for predicting food wastage, with less waste generated from smaller households [34, 36–38]. Moreover, there are studies that found single households to waste the most on a per capita basis [34, 39] as a result of single persons’ lifestyle [32].

### 2.2. Survey design

A survey was conducted to gather information on the socio-economic and behavioral factors that may affect household food waste generation and quantify the composition of this waste in Administrative Beirut, the political and economic capital of Lebanon. The survey is composed of two parts. The first is a questionnaire that gathers information on respondents’ food-related concerns, practices and routines, and perceptions, in addition to sociodemographic characteristics. The second part is a weekly food waste diary that quantifies daily food waste based on a list of 29 food groups classified into four broad categories: (1) bread; (2) home-prepared foods, (3) dairy products, and (4) uncooked and dry foods. For each of the food groups, respondents had to state whether they threw any of it away, and if so, how much and for what reasons.

In order to encourage respondents to participate in filling the food waste diary, they were offered an incentive of USD25 in the form of grocery vouchers upon completion of the diaries. Table 1 displays the four food categories included in the diary with a detailed description of their 29 constitutive food items and their respective measurement units and energy contents. The diary section recording the household food waste (nature, reason, estimated quantity), had to be filled every day for a period of seven days. Finally, to ensure the geographical representativeness of the survey, the number of households to be interviewed in the eight electoral districts of the city was determined in proportion to the number of registered voters in each.

**Table 1:** Sociodemographic characteristics of the respondents.

Sociodemographic	N	Percent/Average
Gender		
Male	11	2.2%
Female	489	97.8%
Household monthly income		
<LBP675,000 (<USD 450)	6	1.2%
LBP676,000—LBP1,200,000 (USD451—USD800)	93	18.6%
LBP1,201,000—LBP2,400,000 (USD801—USD1,600)	181	36.2%
LBP2,401,000—LBP3,599,000 (USD1,601—USD2,399)	135	27.0%
LBP3,600,000—LBP6,749,000 (USD2,400—USD4,499)	63	12.6%
>LBP 6,750,000 (>USD4,500)	6	1.2%
Don't know/Refuse to answer	16	3.2%
Monthly expenditure on food		
<LBP200,000/month	6	1.2%
LBP204,000—LBP396,000	35	7.0%
LBP400,000—LBP596,000	122	24.4%
LBP600,000—LBP796,000	153	30.6%
LBP800,000—LBP1,196,000	167	33.4%
LBP1,200,000—LBP1,996,000	16	3.2%
>LBP2,000,000	0	0.0%
Don't know/Refuse to answer	1	0.2%
Highest level of education		
Elementary (Grade1-6)	19	3.8%
Intermediate (Grade 7–9)	125	25.0%
Secondary/High school (12 years of schooling)	240	48.0%
Some college, but no degree	65	13.0%
University graduate (bachelor degree or equivalent)	30	6.0%
Postgraduate, master`s degree, doctorate	3	0.6%
Technical/vocational	15	3.0%
Refuse to answer	3	0.6%
Number of people living in household		
Total	-	4.03
<6 years old	-	0.13
6–15 years old	-	0.59
16–60 years old	-	2.99
> 60 years old	-	0.32

### 2.3. Data collection

A multi-stage probability sampling approach was adopted to ensure a random, representative sample for identifying households and main respondents. The first stage consisted of representatively selecting neighborhoods inside each district, while the second stage consisted of selecting households based on a systematic random sample in each selected neighborhood according to its estimated number of buildings. Finally, the third stage consisted of sampling a primary respondent within each household. The interviewer asked about the person in charge of food preparation or supervision of the preparation. If the selected person was not at home, a follow-up, up to one time, was conducted before declaring a non-response.

Data collection proceeded over two phases in order to accommodate seasonal variability in food waste patterns. In the first phase, 350 respondents were interviewed, using the questionnaire, in April and October 2016. Out of those, 100 respondents were given seven diary sheets that they had to fill on a daily basis over a period of a week. In the second phase, an additional 150 respondents completed both the questionnaire and diary during October of the same year. On average, respondents needed around 35 minutes to complete the questionnaire. The sociodemographic characteristics of the respondents are shown in Table 1.

This survey was approved for ethical compliance by the American University of Beirut’s Institution Review Board (IRB). In order to ensure the anonymity of the respondents, no personal identifiers (e.g., names, addresses, and phone numbers) were collected. The study was completely voluntary, and participants were also given the choice to quit at any time and refrain from answering any question(s). When necessary, items in the questionnaire were explained to ensure accurate responses.

### 2.4. Estimation of weight and nutrient loss

Several food items included in the diary were composite dishes such as stews, salads, and ready meals. In order to identify the measurement unit and energy content of the food items included in the diary, a representative food or the average of several representative foods was adopted, based on data available from Nasreddine [40]. To assist the respondent in estimating the quantities of food waste, some of the food items’ measurement units were not expressed in kilograms but rather in common household measures such as plate, bottle, can or tablespoon. These were later converted to kilograms as per the units’ typical weights presented in the table. The derived weights were then converted into energy (kcal per 100 grams) values listed in Table 2. A similar procedure was applied for macronutrients and selected micronutrients–including Kilocalories (kcal), Protein (g), Carbohydrate (g), Total Fat (g), Cholesterol (mg), Saturated Fat, Monounsaturated Fat (g), Cholesterol (mg), Potassium (mg), Saturated Fat (g), Monounsaturated Fat (g), Sodium (mg), Calcium (mg), Iron (mg), Dietary Fiber (g), Beta-Carotene, Magnesium (mg), Zinc (mg), Vitamin A (RE), Vitamin C (mg), Vitamin D (μg). (*A table with the nutritional contributions of each of the 29 food items can be provided upon request*). The energy and nutrient content of the food items were estimated based on the Nutritionist Pro Software version 7.1.0 (Axxya Systems, USA). Household daily losses in energy were computed by summation of the respective products of the quantity lost and the energy per gram value for each food item, divided by 7 (since the collected data on food waste was for a period of one week). The same procedure was adopted to determine the household daily loss of macro- and micronutrients. Per capita daily nutrient loss was then obtained by dividing the above-obtained values by 3.948, the average number of occupants per household.

**Table 2:** Food items included in the food waste diary as well as their measurement units and energy content in kcal per 100g.

Food Item	Measurement unit	Energy contribution (kcal/100g)
*Bread*		
Standard/specialty bread	Loaf (120g)	270.5
*Homemade foods*		
Stews	Medium plate (245g)	101.7
Fried foods	Medium plate (145g)	282.3
Salads	Medium plate (200g)	63.3
Cooked starchy foods	Medium plate (154g)	128.4
Roasted/grilled foods	Medium plate (100g)	287
Condiments	Tablespoon (15g)	426.3
Ready meals/ takeaway (Dish)	Medium plate (163g)	274.5
Ready meals/ takeaway (Sandwich)	Sandwich (222g)	245.2
Canned foods	Can (230g)	113.5
*Dairy and eggs*		
Milk (liquid)	Cup (244g)	61
Milk (powder)	Tablespoon(8 g)	134
Dairy products	Serving (112 g)	299.8
Ayran yogurt	Bottle (300 g)	35.5
Eggs	Egg (50 g)	113.8
*Uncooked dry foods*		
Rice	1kg (1000g)	360
Pasta	1kg (1000g)	371
Breakfast cereals	Cup (28 g)	360
Grains	1kg (1000g)	345
Fresh fruits	Medium-sized fruit (143 g)	62.7
Fresh Vegetables	Medium-sized vegetable (53 g)	38.8
Raw/cooked chicken	1kg (1000g)	120
Raw/cooked meats	1kg (1000g)	132
Raw/cooked pork/ham/bacon	1kg (1000g)	748
Raw/cooked fish	1kg (1000g)	72
Drinks (fresh juice/ carbonated soft drink/mineral water)	Cup/can (275 g)	151.4
Deserts	Item (29 g)	392.3
Oil	1kg (1000g)	883.2

### 2.5. Empirical model

In order to investigate the determinants of food waste and nutritional losses, we chose to explain (1) food weight wastes (kg), and (2) caloric losses (kcal), by the set of all variables that we have found in the literature to be related to food waste generation. Indeed this entailed that the quantities reported in the food diary first be translated into kg and kcal, and then regressed on explanatory variables. Not all of the variables mentioned earlier as potential determinants of household food waste have shown significance when included in the regression. The final model used in this paper is the following:

$$Waste=\alpha +\beta_{1}own+\beta_{2}foodexp+\beta_{3}hhsize+\beta_{4}inc+\beta_{5}eatprep+\beta_{6}plan+\beta_{7}offer+\beta_{8}shoplist+\epsilon$$

All details on how the variables were measured and included in the model are shown in Table 3. Given the large proportion of left censoring at zero of the collected data and their panel structure (seven daily observations per respondent), a Tobit model with random effects (RE) was deemed necessary to avoid biased estimates. Both the weight and the caloric loss data were regressed on the same set of explanatory variables. In this case, the Tobit model is designed to estimate the linear relationship between behavioral and sociodemographic variables and the left censoring in the dependent variable, which is weight or energy of food wasted over a seven day period. Based on the Tobit estimates, we also estimated the adjusted mean weight/energy value for each of the covariates’ levels evaluated at the mean of the remaining covariates. This allows us to conduct Wald tests to find out which covariates’ levels are significantly different in terms of their observed mean effect.

**Table 3:** List of variables included in the data analysis and their corresponding descriptions.

Variable (*label*)	Original question and answers	Recorded variables included in the final model
Sole owner (*own*)	*What best describes your status in the current premise*? 1. Sole owner 2. Renting furnished 3. Renting unfurnished 4. Free (family-owned/no rent paid)	Dummy variable denoting if the respondent is ‘Sole owner’ (1). The remaining levels were used as a baseline.
Food expenditure (*foods*)	*How much do you usually spend on food on a monthly basis*? 1. Less than LBP 200,000 2. Between LBP 200,000 and LBP 399,000 3. Between LBP 400,000 and LBP 599,000 4. Between LBP 600,000 and LBP 799,000 5. Between LBP 800,000 and LBP 1,199,000 6. Between LBP 1,200,000 and LBP 1,999,000 7. LBP 2,000,000 or more 99. Don’t know/Refuse to answer	Two dummy variables indicating food expenditures of LBP400,000-LBP799,000 (3–4) and ≥LBP800,000 (5–7), respectively. Food expenditures of less than LBP 400,000 (1–2) were used as baseline. Note that no respondents reported that they didn’t know their actual expenditure or refused to answer (99).
Total number of people in household (*size*)	*How many persons live in your household*, *including you and the domestic helper(s)*?	Two dummy variables indicating that the number of occupants was 2–3 and more than 3, respectively. Households of 1 occupant were used as baseline.
Household income (*inc*)	*What is the combined monthly income of all persons living in your household*? 1. Less than LBP 675,000 2. Between LBP 676,000 and LBP 1,199,000 3. Between LBP 1,200,000 and LBP 2,399,000 4. Between LBP 2,400,000 and LBP 3,599,000 5. Between LBP 3,600,000 and LBP 6,749,000 6. LBP 6,750,000 or more	Two dummy variables indicating household incomes of LBP1,200,000-LBP2,399,000 (3) and ≥LBP2,400,000 (4–6), respectively. Incomes of <LBP1,200,000 (1–2) were used as baseline.
Eat everything prepared (*eatprep*)	*How often do you eat everything you prepare/ serve*? 1. Hardly ever 2. Rarely 3. Sometimes 4. Frequently 5. Regularly 99. Don’t know	A dummy variable indicating “Frequently/Regularly” (4–5). “Sometimes at most” (1–3) was used as baseline. Note that no respondents answered “Don’t know” (99).
Plan ahead and buy only what I came for (*plan*)	*Please indicate how much you agree with the following statement*: *"When I go grocery shopping I always plan ahead and buy only what I came for"* 1. Strongly disagree 2. Disagree 3. Neither agree nor disagree 4. Agree 5. Strongly agree	A dummy variable indicating “Agree” (4–5). “Disagree/Neither” (1–3) was used as baseline.
Buy special offer (*offer*)	*Please indicate how much you agree with the following statement*: *"When I go grocery shopping I often buy things by impulse (items on sale/special offer) (eg buy one get one free)"* 1. Strongly disagree 2. Disagree 3. Neither agree nor disagree 4. Agree 5. Strongly agree	A dummy variable indicating “Agree” (4–5). “Disagree/Neither” (1–3) was used as baseline.
Prepare a shopping list before going to store (*shoplist*)	*Please indicate how much you agree with the following statement*: *"I usually prepare a shopping list before I head to the grocery store"* 1. Strongly disagree 2. Disagree 3. Neither agree nor disagree 4. Agree 5. Strongly agree	A dummy variable indicating “Agree” (4–5). “Disagree/Neither” (1–3) was used as baseline.

## 3. Results

### 3.1. Caloric and nutrient losses associated with household food waste generation

Table 4 shows the daily per capita losses in terms of energy, macro- and micronutrients in urban Lebanon. Caloric loss was estimated at approximately 451 kcal/day. The food groups that had the highest contribution to calorie loss were dairy products, oil and bread (17.2, 12.2 and 8.7 percent; respectively). The contribution of the various macronutrients to energy loss was estimated at 54.7 percent for fat, 33.2 percent for carbohydrates and 13.2 percent for protein (Table 4). As for dietary fiber waste, it was estimated at approximately 3 g/day (Table 4), with grains being the main contributor to this waste (48.2 percent). The loss of calcium was estimated at 165.2 mg/day (Table 4) and the results showed that 61.7 percent of calcium loss came from the wastage of dairy products. Potassium and Magnesium losses were estimated at 343.2 and 43.3 mg/day, respectively. As for the micro minerals iron and zinc, their losses were in the range of 1.45–1.7 mg/ day. Waste estimates pertinent to three vitamins are also shown in Table 4 (163.9 RE for vitamin A; 8 mg for vitamin C and 2.4 μg for vitamin D per day). Furthermore, Table 5 provides a summary of the types of foods wasted per household per week. More on the interpretation of these results in the discussion section below.

**Table 4:** Daily per capita nutrient loss from 29 food items at the consumer level.

Nutrient	Nutrient loss per capita per day in Administrative Beirut
**Energy, macronutrients, and fiber**	
Energy (kcal)	451.2
Carbohydrates (g)	37.5
Protein (g)	14.9
Total fat (g)	27.4
Cholesterol (mg)	46.7
Saturated fat (g)	8.3
Mono-saturated fat (g)	9.5
Dietary Fiber, Total (g)	2.9
**Minerals**	
Calcium (mg)	165.2
Iron (mg)	1.45
Magnesium (mg)	43.3
Zinc (mg)	1.7
Potassium (mg)	343.2
**Vitamins**	
Vitamin A (RE)	163.9
Vitamin C (mg)	8.0
Vitamin D (μg)	2.4

**Table 5:** Food waste by type (kg per household per week).

Food Type	Mean	Std. Dev.	Min	Max
Standard/specialty bread	0.680	0.433	0.120	2.400
Stews	0.849	0.535	0.245	3.920
Fried foods	0.448	0.262	0.145	1.740
Salads	0.654	0.402	0.200	2.400
Cooked starchy foods (pasta, potatoes)	0.519	0.288	0.154	1.848
Roasted/grilled foods	0.336	0.231	0.100	1.200
Condiments (ketchup, mayonnaise, mustard, etc.)	0.034	0.022	0.006	0.060
Ready meals/ takeaway (plates/dishes)|	0.535	0.311	0.163	1.304
Ready meals/ takeaway (sandwiches)	0.770	0.471	0.222	2.664
Milk (liquid)	0.541	0.500	0.244	3.904
Milk (powder)	0.019	0.013	0.008	0.064
Dairy products (cheese, strained yogurt (labneh), yoghurt, butter, etc.)	0.245	0.185	0.112	1.344
Yoghurt drink (Ayran)	0.931	0.692	0.300	3.600
Eggs	0.305	0.220	0.050	1.200
Rice	2.625	1.885	1.000	8.000
Breakfast cereals	5.193	0.999	2.240	5.600
Fresh fruits	0.251	0.170	0.143	1.144
Fresh vegetables	0.093	0.069	0.053	0.424
Raw/Cooked chicken	2.425	1.466	1.000	8.000
Raw/Cooked meats (beef, veal)	2.095	1.462	1.000	9.000
Raw/Cooked pork/ham/bacon	2.636	1.706	1.000	8.000
Raw/Cooked fish	1.864	0.834	1.000	4.000
Beverages (fresh juice/ carbonated soft drink/mineral water)	0.807	0.592	0.275	3.300
Deserts (candy, cake, etc.)	0.040	0.015	0.029	0.087
Oil (vegetable oils, olive oil, etc.)	1.933	0.000	1.000	4.000

### 3.2. Determinants of household food waste generation

Descriptive statistics of the variables included in the empirical model are presented in Table 6. Results of the Tobit model estimating the weight of food waste (kg) and caloric losses (kcal) are presented in Table 7 and Table 8; respectively. The coefficients in Tables 7 and 8 reflect the direction, magnitude, and significance of the change in the dependent variable’s value associated with any level of a given covariate relative to that covariate’s base level. We further manipulate these coefficients in order to derive the absolute mean expected values of weight and caloric wastes, as presented in the same tables.

**Table 6:** Summary statistics of the various variables included in the Tobit model.

Variable	Observations	Percentages
***Sole owner***		
No	1,750	54.0%
Yes	1,750	46.0%
***Food expenditure***		
<LBP400,000	1,750	10.4%
LBP400,000-LBP799,000	1,750	63.2%
≥LBP800,000	1,750	26.4%
***Total number of people in household***		
1 occupant	1,750	1.6%
2–3 occupants	1,750	28.4%
>3 occupants	1,750	70.0%
***Household income***		
<LBP1,200,000	1,750	19.2%
LBP1,200,000-LBP2,399,000	1,750	35.2%
≥LBP2,400,000	1,750	40.4%
Don't know	1,750	5.2%
***Eat everything prepared***		
Sometimes at most	1,750	36.0%
Frequently/Regularly	1,750	64.0%
***Plan ahead and buy only what I came for***		
Disagree/Neither	1,750	39.6%
Agree	1,750	60.4%
***Buy special offers***		
Disagree/Neither	1,750	48.0%
Agree	1,750	52.0%
***Prepare a shopping list before going to store***	*** ***	
Disagree/Neither	1,750	52.4%
Agree	1,750	47.6%

**Table 7:** Tobit model estimates for food waste (kg).

Variable	Coefficient	z	Expected weight waste (kg)	Group comparisons*	
				5%level	10% level
***Sole owner***					
No	0 (base)	-	0.757		
Yes	0.403	3.18	1.047		
***Monthly food expenditure***					
<LBP400,000	0 (base)	-	0.694	A	A
LBP400,000-LBP799,000	0.197	0.87	0.824	A	A
≥LBP800,000	0.604	2.22	1.128		
***Total number of people in household***					
1 occupant	0 (base)	-	0.26		
2–3 occupants	1.124	2.05	0.842	A	A
>3 occupants	1.237	2.27	0.923	A	A
***Household income***					
<LBP1,200,000	0 (base)	-	1.252		
LBP1,200,000-LBP2,399,000	-0.547	-2.91	0.832	A	A
≥LBP2,400,000	-0.618	-2.81	0.784	A	A
Don't know/Refuse to answer	-0.576	-1.86	0.812	A	A
***Eat everything prepared***					
Sometimes at most	0 (base)	-	1.059		
Frequently/Regularly	-0.363	-2.42	0.794		
***Plan ahead and buy only what I came for***					
Disagree/Neither	0 (base)	-	0.7		
Agree	0.454	3.36	1.018		
***Buy special offers***					
Disagree/Neither	0 (base)	-	0.996	A	
Agree	-0.288	-1.88	0.789	A	
***Prepare a shopping list before going to store***					
Disagree/Neither	0 (base)	-	0.807	A	A
Agree	0.235	1.55	0.976	A	A
Constant	-0.509	-0.83	-		

Groups sharing a letter have expected weight losses that are not significantly different at the specified significance levels.

**Table 8:** Tobit model estimates results for caloric losses (kcal).

Variable	Coefficient	z	Expected caloric loss (kcal)	Group comparisons*	
				5% level	10% level
***Sole owner***					
No	0 (base)	-	1301.51		
Yes	928.81	2.84	1817.48		
***Monthly food expenditure***					
<LBP400,000	0 (base)	-	1116.90	A	A
LBP400,000-LBP799,000	715.47	1.22	1471.43	A	A
≥LBP800,000	1842.28	2.62	2154.36		
***Total number of people in household***					
1 occupant	0 (base)	-	664.63		
2–3 occupants	2898.21	2.03	2069.57	A	A
>3 occupants	3210.45	2.27	2280.05	A	A
***Household income***					
<LBP1,200,000	0 (base)	-	2100.46	B	B
LBP1,200,000-LBP2,399,000	-1173.89	-2.41	1403.45	A	A
≥LBP2,400,000	-1323.83	-2.33	1326.32	A	A
Don't know/Refuse to answer	-1137.59	-1.42	1422.54	AB	AB
***Eat everything prepared***					
Sometimes at most	0 (base)	-	1739.17	A	A
Frequently/Regularly	-669.26	-1.72	1367.32	A	A
***Plan ahead and buy only what I came for***					
Disagree/Neither	0 (base)	-	1295.29		
Agree	953.75	2.73	1825.10		
***Buy special offers***					
Disagree/Neither	0 (base)	-	1682.60	A	A
Agree	-477.55	-1.20	1417.26	A	A
***Prepare a shopping list before going to store***					
Disagree/Neither	0 (base)	-	1423.72	A	A
Agree	453.05	1.15	1675.46	A	A
Constant	-2116.32	-1.33	-		

Groups sharing a letter have expected caloric losses that are not significantly different at the specified significance levels.

In terms of sociodemographic determinants, being the sole owner of the residence shows a positive and significant impact on food waste generation in comparison to non-owners, mainly tenants. The model suggests that it is associated with an average of 1.05 kg of daily household food waste, nearly 0.3 kg higher than non-owners are. Moreover, being the sole owner of a residence increases the amount of food energy wasted by around 500 kcal compared to tenants.

The impact of homeownership—which largely reflects higher income in the context of Lebanon—on food waste generation, resonates with that of food expenditure, whereby increasing levels appear to have a positive and significant impact on the amount of food wasted. It suggests that households that spend the most on food (more than LBP 800,000 (US $533) per month) waste between 0.20 and 0.45 kg more food per day than those who spend less, all else being constant. Additionally, the positive coefficient for food expenditure in the caloric loss model implies that households which spend at least LBP400,000 (US $267) per month on food waste, between 700 and 1000 kcal more per day than households who spend less than that.

As for the number of members in the household, the model suggests that it increases the amount of food wasted. Having two or more occupants in the household significantly enhances food waste generation by at least 0.58 kg daily per household. In addition, the model for caloric losses shows that the amount of food energy wasted in households with two occupants or is at least 1,400 kcal more than households with single occupancy are. Turning to income, results obtained in our model show that households earning LBP 1,200,000 (US $800) or more per month generate at least 0.4 kg less waste in comparison with those less than LBP 1,200,000. The caloric loss model suggests that households with a combined income of LBP 1,200,000 (US $800) per month or higher waste at least 700 kcal less per day as compared to households with a monthly income less than LBP 1,200,000.

Regarding behavioral variables included in the analysis, respondents who “frequently” eat everything they prepared waste on average 0.26 kg less than those who at most “sometimes” eat everything they prepared, but the differences for caloric waste appear to be insignificant. Buying special offers also has a significant negative effect (at the 10 percent level) on household food waste generation, whereby respondents who either claim not to buy special offers or are unsure about this waste around 0.21 kg more food per day compared to respondents who say they often buy special offers. Again, the differences in caloric losses across the two groups are not significant. Finally, though the effect of preparing a shopping list seems to result in slightly higher food waste, this result remains insignificant even at the 10 percent level, hence providing no evidence that this type of behavior reduces food waste.

## 4. Discussion

This study has provided the first estimate of energy and nutrient losses associated with household food waste generation in Lebanon. Caloric loss was estimated at approximately 451 kcal/day, with dairy products, oil, and bread being the main contributors to this caloric loss. The magnitude of energy loss observed in this study is higher than that reported in Turkey (246 kcal/day); whilst being lower than levels described in the US (759–789 kcal/day) [17, 19, 41].

In agreement with our findings, a study conducted by Thonissen [42] highlighted dairy products as the largest contributors to energy loss in the Netherlands, and Buzby, Farah-Wells [19] have reported oil amongst the three major contributors to caloric loss in the US (19.5 percent). In the present study, fat represented close to half of caloric loss, while carbohydrates and protein represented 33.2 percent and 13.2 percent, respectively. Our findings are in agreement with those reported for the US, where fat loss has also contributed close to half of energy loss. In this regard, it is important to note that focusing only on the caloric value and macronutrient content of wasted food can over-represent the influence of energy and fat-dense foods, losing sight of other essential nutrients that are lost (Spiker et al, 2017). Nutrient-dense foods such as dairy products, fruits, vegetables, and fish are wasted at disproportionately high rates, implying that food waste may have a significant impact on the supply of essential nutrients such as micronutrients and dietary fiber (Spiker et al, 2017). Hence, in our study, we have also evaluated the nutritional content of food waste in terms of dietary fiber and micronutrients.

The waste of dietary fiber was estimated at approximately 3 g/day, a value that is approximately half the estimate reported by Spiker et al (2017) in the US (5.9 g). However, it is important to note that the amount of total food waste reported by Spiker et al (2017) was more than double the amount observed in our study (1216 kcal vs. 451 kcal) which may explain the higher dietary fiber loss described in their study. The loss of dietary fiber should be viewed as a concern given that available evidence indicates that two-third of Lebanese adults do not meet the recommended intake levels of dietary fiber (Nasreddine et al, unpublished data). Adequate fiber intake, which is estimated to range between 19 and 38 grams per day depending on age and gender [43], was repetitively shown to decrease the risk of chronic diseases, and the Academy of Nutrition and Dietetics recommends consuming fiber in its food form, and not as dietary supplements [44]. As Lebanon harbors one of the highest burdens of chronic diseases in the Eastern Mediterranean region, with close to 84 percent of mortality being attributed to these diseases, the findings of this study reveal the importance of minimizing the loss of dietary fiber as a cost-effective public health strategy [45].

Our study has also importantly documented the losses in several micronutrients including iron, calcium, and vitamin D. These losses were expressed on a per capita basis and, as such, may not reflect the actual losses that may occur at the individual level within the household, particularly amongst the nutritionally vulnerable individuals such as the elderly, women of reproductive age and young children. These micronutrients (iron, calcium and vitamin D) have been repetitively flagged as a public health concern in Lebanon [46] [47], given their suboptimal consumption levels, in comparison with the dietary reference intakes (7–18 mg/day for iron, 700–1300 mg/day for calcium, and 15–20 μg/day for vitamin D) [48]. Few other studies have investigated micronutrient losses resulting from food waste (Spiker et al, 2017). Although the loss of iron observed in our study (1.45 mg/day) is lower than the value reported for the US (5.3 g/day), this loss remains a public concern given that iron deficiency and anemia are highly prevalent in the country. [49–51]. Similarly, our findings showed that the loss of calcium was estimated at 165 mg/d and that this loss was mostly due to dairy wastage (61.7 percent). In agreement with our results, Spiker et al (2017) have shown that 72 percent of calcium loss in the US was due to the waste of dairy products. Previous studies conducted in Lebanon have reported low dietary intake of calcium, particularly among children and women of reproductive age [52–54].

The observed loss of energy, macro- and micronutrients should be viewed as an opportunity for culture-specific interventions aimed at decreasing food waste at the consumer level, particularly given the rising threat of food insecurity in the country. Recent studies conducted in rural, semi-urban and urban regions of Lebanon showed that household food insecurity is quite prevalent, reaching 34, 42 and 50 percent in the Bekaa, South and Greater Beirut regions; respectively [55–57]. The study findings should stimulate policymakers, food, and health professionals, as well as nutritionists, to develop intervention strategies aimed at reducing food waste at the consumer level. However, these findings should not be interpreted as denoting that all lost nutrients could be recovered and fed to the consumer, nor that the recovered food would necessarily result in an attractive, palatable or nutritious diet (Spiker et al, 2017). Some food will unescapably be discarded, particularly in light of food safety concerns that remain a priority when dealing with perishable foods (Spiker et al, 2017). Even though only a portion of the lost nutritional content can be recovered for human consumption, the magnitude of food waste and the associated loss in nutrients, money, and resources imply that there is an opportunity in focusing on that effort (Spiker et al, 2017).

Turning to the empirical models of the drivers of household food waste generation, our results suggesting that sole owners waste more food than non-owners is contrary to evidence from the UK, where homeowners have been found to waste less food than tenants [58]. This can be partly explained by the fact that those who own their homes are from older generations and by the likelihood that their houses would be smaller. However, this claim could not be held true in a country like the United States where owned residences feature greater house areas, supposedly leading to a higher generation of waste [11]. In Lebanon, residential tenants are not necessarily younger, since a large proportion of lower-income households benefit from a highly secure and cheap ‘old tenancy law’–This law was enacted after World War II to prevent socio-economic deprivation and protect tenants from greedy property owners, the law resulted in the saying, “the tenant is an owner”, as it gave many rights to the renter. Under the law, all rental contracts would be extended–against the will of the landlord–until a new one was enacted, meaning rents were fixed at the originally agreed upon rate despite inflation and changes in market dynamics.

As for the effect of household size, results derived from the model are in line with the literature, where evidence suggests that larger household sizes, particularly with younger members, have a higher effect on waste generation [59, 60]. Regarding income, as mentioned earlier, some of the studies conducted found a positive relationship between income and food waste generation whereas other studies did not find any effect. The results obtained in our model diverge from previous findings and show a negative correlation between income and food waste. One possible explanation could be that high-income earners are more likely to possess better conditions of food storage, which will help them conserve food for longer periods, especially as they have better access to private electricity generation in the context of frequent electricity blackouts in Lebanon.

When it comes to variables related to household behavior related to food, results from the model show significant effects of “eating everything prepared”, “planning ahead and buying only what one came for”, “buying special offers”, and “preparing a shopping list before going to store”. The models show that those who report eating everything prepared generate less food waste, a result that is consistent with studies in the literature [33]. As regards “buying special offer”, the model reveals a negative correlation with food waste. In fact, numerous studies show that food waste is lower on average in households that have a propensity to buy discounted foods [34, 39]. As for “planning ahead and buying only what one came for” and “preparing a shopping list before going to the store”, results from the models show a positive effect of these two planning practices on food waste generation, in contrast with results reported in the literature [27]. One possible explanation for this result could be that planning and preparing shopping lists may lead to buying food more than needed, or overprovisioning [9]. Overprovisioning has been ascribed to many factors such as “the good provider identity”, which defines someone’s wish to be a good parent or a good partner, “differences in taste” and “oversized packaging” [23]. So even when preparing a shopping list and sticking to it when shopping for food, many items on such lists that could be considered as “surplus” and hence lead to food waste.

## 5. Conclusions

This study has provided the first estimate of energy and nutrients’ losses affected by household food waste generation in Lebanon. This analysis has shed light on the main socio-economic and behavioral contributors to food waste at the household level. In a country where the threats of food insecurity and hidden hunger are on the rise, particularly amongst vulnerable population groups, the results of the study should stimulate policymakers, food, and health professionals, as well as nutritionists, to develop intervention strategies aiming at reducing food waste at the consumer level. Furthermore, different avenues for preventing food waste should be explored to help alleviate hunger and malnutrition. Such actions should be part of a strategy addressing the environmental challenges pertinent to food waste and its disposal, mainly landfills that are increasing the burden on key natural resources and affecting population health significantly. Main interventions may include consumer education and awareness strategies, as well as regular tracking of products’ expiry dates, all of which are cost-effective in the long run [61]. The estimates obtained in this study may also be used as a baseline to help evaluate the efficiency and outcome of such future interventions on food waste in Lebanon.

The current findings should be interpreted in light of several limitations. First, some of the drivers that have been found to be key in food waste according to prior research were not included in the final models. In the case of age, this variable was included in various earlier specifications of the models and yet was found to be consistently insignificant. On the other hand, access to private electricity generation was also not included in the model as we failed to collect information about this aspect in the survey questionnaire.

Furthermore, the estimated losses in energy and nutrient content were expressed on a per capita basis and, as such, may not reflect the actual losses that may occur at the individual level within the household, particularly amongst the nutritionally vulnerable individuals such as the elderly, women of reproductive age and young children. Moreover, this study relies on approximations of edible food quantities lost across a large number of categories, and as such is reliant on more than one layer of assumptions. Indeed, it provides rough first-cut estimates of food waste across various nutrients and reveals very interesting insights on the sociodemographic and behavioral drivers of household food waste. Finally, a limitation in our method is its reliance on self-reported food waste, with its potential to generate downwardly biased results due to systematic underestimation of waste levels [62].

In conclusion, little is known about household food waste generation in developing countries in general and Lebanon in particular, and the findings of this work and future related studies in this area could help provide evidence and data for decision-makers to address both food waste as well as food and nutrition security issues.
